# Distance sampling for epidemiology: an interactive tool for estimating under-reporting of cases from clinic data

**DOI:** 10.1186/s12942-020-00209-1

**Published:** 2020-04-20

**Authors:** Luca Nelli, Moussa Guelbeogo, Heather M. Ferguson, Daouda Ouattara, Alfred Tiono, Sagnon N’Fale, Jason Matthiopoulos

**Affiliations:** 1 University of Glasgow, Institute of Biodiversity Animal Health and Comparative Medicine, Glasgow, UK; 2 Centre National De Recherche et Formation sur le Paludisme, Ouagadougou, Burkina Faso

**Keywords:** Access to health care, Distance sampling, Malaria, Passive surveillance, Reporting bias

## Abstract

**Background:**

Distance sampling methods are widely used in ecology to estimate and map the abundance of animal and plant populations from spatial survey data. The key underlying concept in distance sampling is the detection function, the probability of detecting the occurrence of an event as a function of its distance from the observer, as well as other covariates that may influence detection. In epidemiology, the burden and distribution of infectious disease is often inferred from cases that are reported at clinics and hospitals. In areas with few public health facilities and low accessibility, the probability of detecting a case is also a function of the distance between an infected person and the “observer” (e.g. a health centre). While the problem of distance-related under-reporting is acknowledged in public health; there are few quantitative methods for assessing and correcting for this bias when mapping disease incidence. Here, we develop a modified version of distance sampling for prediction of infectious disease incidence by relaxing some of the framework’s fundamental assumptions. We illustrate the utility of this approach using as our example malaria distribution in rural Burkina Faso, where there is a large population at risk but relatively low accessibility of health facilities.

**Results:**

The modified distance-sampling framework was used to predict the probability of reporting malaria infection at 8 rural clinics, based on road-travel distances from villages. The rate at which reporting probability dropped with distance varied between clinics, depending on road and clinic positions. The probability of case detection was estimated as 0.3–1 in the immediate vicinity of the clinic, dropping to 0.1–0.6 at a travel distance of 10 km, and effectively zero at distances > 30–40 km.

**Conclusions:**

To enhance the method’s strategic impact, we provide an interactive mapping tool (as a self-contained R Shiny app) that can be used by non-specialists to interrogate model outputs and visualize how the overall probability of under-reporting and the catchment area of each clinic is influenced by changing the number and spatial allocation of health centres.

## Background

Estimates of infectious disease incidence at local, regional and national scales are typically based on clinical records of symptomatic cases as reported to the public health system (e.g. from clinics or hospitals). It has long been recognized that estimates of disease burden acquired from such *passive surveillance* will be biased by under-reporting [1–6]. Such problems of under-reporting are heightened in settings where there are significant barriers to health system access [7–10], for example due to an unbalanced geographical distribution of clinics [11], difficult travel routes [12, 13] or socio-economic barriers to health-seeking [10, 14]. Consequently under-reporting is particularly likely in rural areas in low- and middle-income countries where all of these variables may combine to limit access to health services [1, 2, 9, 15–17]. In such settings, disease burden is optimally estimated through community-based surveys [18], or *active surveillance* [5]. Although more accurate, active surveillance programmes are considerably more expensive and time consuming than passive surveillance through the health system, and are thus usually only possible for a few time points at a limited number of locations.

Consequently, burden estimates in low and middle incomes countries are typically derived from passive surveillance for some of the most important infectious diseases, including malaria [18–20] and dengue [6, 21]. An advantage of this system is that it incorporates data from a large number of geographically dispersed clinics, and thus offers opportunity for large-scale spatial predictions. However, although this system may reliably reflect the epidemiological trends, the remarkable levels of underreporting arising from self-reporting at health centres [6, 18] can limit the accuracy of spatial predictions. These biases arising from spatial variation in under-reporting are rarely formally quantified. When estimating the ‘true’ disease incidence in a community, a multiplication factor can be used to adjust values to account for under-reporting [5, 20]. Factors known to influence reporting include the severity of disease symptoms (e.g. probability of asymptomatic infection [22–26]) and sociodemographic factors that encourage or impede self-reporting, such as poverty and education [8, 10, 16, 27, 28], ethnicity [29, 30] and language barriers [31]. However, the estimation of reporting completeness at national level can suffer from systematic biases [20]. For example, utilization of health services in the population is not homogeneous [32]. Formal quantification of the probability of under-reporting can be expressed as a function of the effective distance between the patient’s residence and the nearest health facility [12, 13, 32–40].

Spatial mapping of disease incidence requires thus robust quantification not only of the factors that influence epidemiological risk, but also of those affecting under-reporting [3, 41–43]. Measurement and assessment of the full range of environmental, socio-economic and geographic variables that can impact health-seeking behaviour is difficult to achieve at a population-level. However, one piece of crucial information that is regularly recorded at health facilities is patient residence (either specific address, or community of residence). This information can be used to calculate travel distance between a case and the health clinic, thus providing an opportunity to quantify one of the major causes of under-reporting: distance. Methods to infer detection probability based on the distance between an object and an observer have been formally developed in the distance sampling framework, a well-established methodology used primarily in wildlife ecology to estimate density or abundance of animal or plant populations [44]. The list of practical applications of this method is constantly growing, and encompasses a wide variety of taxa (e.g. http://distancesampling.org). Detailed derivation of these methods can be found in ST Buckland [44], ST Buckland, DR Anderson, KP Burnham, JL Laake, DL Borchers and L Thomas [45] and ST Buckland, EA Rexstad, TA Marques and C Oedekoven [46]. However, so far this method has not been applied to predict infectious disease incidence in humans based on reporting to health systems.

Here, we adapt the conventional distance sampling approach to the estimation of under-reporting of disease, using the example of malaria incidence in a rural setting in Burkina Faso where access to health clinics is limited due to poor road infrastructure, poverty, and seasonal weather events [47–49]. Within this context, we define the clinics where people report as the “observers”, with the event we are trying to detect being malaria infection. Thus, we focus on the subset of distance sampling methods that deal with stationary observers (performing so-called, *point*-*transect* distance sampling).

In their simplest form, point-transect survey methods assume that all occurrences within a predetermined distance *w* from the observer’s position are detected. It is then possible to use these local counts to quantify correlations with geographical covariates, or simply scale them up to estimate the total number of occurrences across the landscape [44]. Distance sampling relaxes the assumption of perfect detectability by introducing the probability that an object within the surveyed area *a* is detected. This probability may decay with distance $d$ from the observer, a property described by the detection function $P(d)$. The detection function can be further improved by the inclusion of covariates other than distance, and is estimated from the observations subject to three key assumptions. First, the zero-distance assumption dictates that $P(0)\, = \,1$ (i.e. the observer cannot miss an occurrence at their exact position). Second, the independence assumption dictates that observers are independent of each other and, in particular, that any given occurrence may be recorded by more than one observer. Third, the Euclidean distance assumption postulates that detection varies as a function of straight-line distance on a Cartesian system of coordinates.

Extending the application of the point-transect distance sampling method to clinic data requires us to relax its three key assumptions, by acknowledging that cases may go undetected even at zero distance (e.g. asymptomatic cases), that after reporting at one clinic a patient, will not report elsewhere (non-independence of observers), and that detection may vary as a function of non-Euclidean distance measures, related to road-network or other determinants of accessibility.

Here, we begin the process of adapting distance sampling methods for epidemiological prediction by presenting the fundamental concepts for estimation of a detection function for clinic data, implementing them within a Bayesian framework of statistical inference and illustrating their use through a case study of malaria reporting in rural south-western Burkina Faso. This area of Africa experiences a particularly high burden of malaria [50, 51], creating an urgent need for accurate prediction of incidence. Finally, to illustrate how our approach could be used for public health planning, we present an interactive mapping tool (R Shiny app), built upon the model results from the malaria case-study. This can be used by non-specialists to interrogate model outputs and visualize how the overall probability of under-reporting and the individual clinic catchment area is influenced by changing the spatial distribution of health centres.

## Methods

### Statistical analyses

For a given geographical area of interest, out of $N$ actual clinics we consider a subset of $J$ participating clinics. We consider a dataset comprising of a list of patients $i \in \{ 1, \ldots I\}$, each reporting at one of the $J$ participating clinics. We develop inference for the subset of cases that are reported to participating clinics because, by definition, all other reported cases will not be found in the data set. For each $i\text{th}$ patient reporting at any one of $J$ participating clinics, we define a data vector of “clinic reporting choice” $h_{i} \, = \,\{ h_{i,1} , \ldots ,h_{i,J} \}$ of length $J$, with value 1 at the $j\text{th}$ clinic, where the case was reported, and 0 in all the other cases. Such data can be described as realisations from a single-trial multinomial process,

1 $$\varvec{h}_{i} \sim Multinomial\left( {1, \varvec{P}_{\varvec{i}} } \right)$$

where the likelihood of a positive outcome (disease case being reported) at the $\text{jth}$ clinic is determined by the vector of probabilities  $\varvec{P}_{i} = \left\{ {P_{i,1} , \ldots , P_{i,J} } \right\}$ of reporting the $i\text{th}$ case at the $i\text{th}$ clinic.

Any given case may be reported to any one of the participating clinics, but nearby clinics are more likely to receive the report. Under these assumptions, the probability of any one case being reported to any one clinic (accounting for other clinics) can be modelled in terms of the distances of all the clinics from the point of occurrence of the case, as follows:

$$P_{i,1} = \frac{{g_{i,1} }}{{1 + \mathop \sum \nolimits_{j = 1}^{J - 1} g_{i,j} }}$$

$$P_{i,2} = \frac{{g_{i,2} }}{{1 + \mathop \sum \nolimits_{j = 1}^{J - 1} g_{i,j} }}...$$

2 $$P_{{i,\left( {J - 1} \right)}} = \frac{{g_{{i,\left( {J - 1} \right)}} }}{{1 + \mathop \sum \nolimits_{j = 1}^{J - 1} g_{i,j} }}$$

where $g_{{i,\left( {1, \ldots , J - 1} \right)}}$ represents the decay in the probability of reporting a disease case and is expressed as a function of distance $g\left( {d_{i,j} } \right)$ between the place of residence of the $i\text{th}$ patient and the location of the $j\text{th}$ clinic. In traditional distance sampling approaches, the detection probability may be formulated as a half-normal (HN), a hazard rate (HR) or a negative exponential (NE) model:

3 $$\begin{aligned}{HN:g( d)} = { \exp }\left( {\frac{{ - d^{2} }}{{2\sigma^{2} }}} \right).\\ HR:g\left( d \right) = 1 - { \exp }\left[ { - \left( {\frac{d}{\sigma }} \right)^{ - \beta } } \right]. \\ NE:\,g(d)\, = \exp \,\left( {\frac{ - d}{\sigma }} \right)\end{aligned}$$

where α and β are shape parameters, describing the rate of decay in detection probability with increasing distance. These three models are generally good options for traditional distance sampling, however they are not all appropriate for the proposed multinomial process because, following normalisation of probabilities to 1, a case reported at the exact location of a particular clinic could not receive a probability of 1. For this reason, we introduce a generalised formulation of these standard models:

4 $$g\left( d \right) = { \exp }\left( {a_{0} + a_{1} d^{c} } \right)$$

This function collects the main features of the functions in Eq. (3), such as the exponential behaviour of HN and NE and the exponent for the decay rate in HR. However, we add the estimation of an intercept $a_{0}$ which is extracted from data on the behaviour of the detection function at zero distance and allows the function to work under the normalisation proposed (the multinomial process of Eq. (2)), therefore allowing relaxation of the independence-of-observations assumption.

Health care accessibility needs to take into account both spatial and non-spatial factors, such as demographics or socioeconomic status [7, 8, 16, 52–55] that influence health seeking behaviour. To demonstrate that this model can be extended to include other non-spatial predictors of disease reporting, we tested two other models that included biologically realistic correlates of disease reporting: age of the patient (A), sex (S):

5 $$g\left( {d,A} \right) = { \exp }\left( {a_{0} + a_{1} d^{c} + a_{2} A + a_{3} Ad} \right)$$

6 $$g\left( {d,S} \right) = { \exp }\left( {a_{0} + a_{1} d^{c} + a_{2} S + a_{3} Sd} \right)$$

Poor road conditions can increase travel time and reduce health-seeking behaviour. This is particularly true in rural Africa, where road conditions are strongly weather dependent. To account for that, we tested another model that included season (R) as a categorical variable (wet or dry season).

7 $$g\left( {d,R} \right) = { \exp }\left( {a_{0} + a_{1} d^{c} + a_{2} R + a_{3} Rd} \right)$$

## Malaria case study

### Study area and data collection

The distance sampling model described above was applied to a case study of malaria incidence quantification in rural Burkina Faso. Burkina Faso has one of the high rates of malaria in Africa [50, 51], with the bulk of transmission occurring in rural areas during or shortly after the rainy season between July to December. The primary level of the national health system is constituted by a network of health centres (*centre de santé et de promotion sociale*, CSPS). Each health centre covers several villages (approximately 1 centre for 10,000 habitants) and they represent the first-line of points of contact with the population, in terms of disease diagnosis and treatment. However, access to these clinics can be limited due to poor road infrastructure, poverty, and seasonal weather events [47–49].

**Fig. 1:**
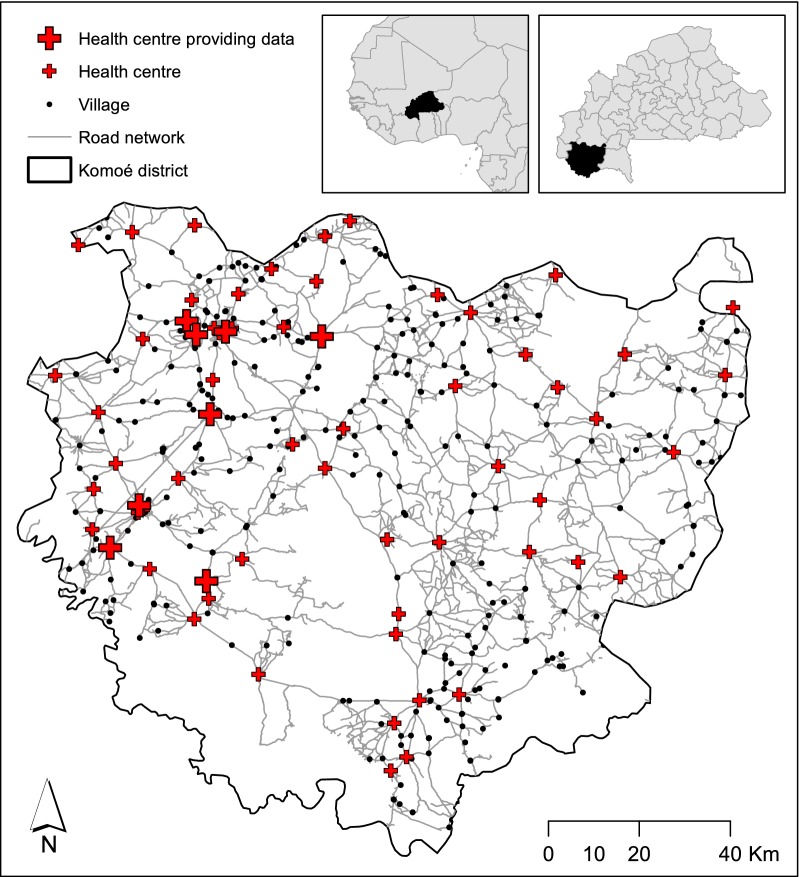
Distribution of health centres (red crosses), villages (black dots) and road network (grey lines) in Komoé district (solid line) within Burkina Faso (insets)

We used data on malaria cases as reported at 8 clinics in the Komoé district, in south-western Burkina Faso (Fig. 1) between January and December 2017. This is a rural area that consists primarily of West Sudanian savannah, made up of 234 discrete village communities with a total censused population of 428,019 in 2016 (mean per village 1829 ± 1915 dev. std., Institut national de la statistique et de la démographie, *unpublished data*). Most of the road network comprises secondary and tertiary roads, with difficult access during the rainy season. This area comprises 64 clinics, that are the first point of contact for communities seeking malaria diagnosis and treatment. Clinics in our study are managed by the same health authority, and therefore share a common operational timetable, quality of infrastructure and equipment, diagnostic capabilities and availability of drugs. Clinical data at these facilities are recorded in a logbook implemented by the national health information system (*système national d’informations sanitaires*, SNIS) and are monthly summarized and transmitted to the national level.

From the registers of each individual clinic, we obtained the permission (Ministry of Health National Centre of Research and Training on Malaria—*Centre National De Recherche et Formation sur le Paludisme*, CNRFP) to retrospectively extract the list of anonymized individual cases of patients (both adult and children) reporting from January to December 2017 a malaria episode, confirmed by rapid diagnostic test (RDT). For each individual case we extracted the following key data: consultation date, age, sex and reported village of origin. Anonymized data were recorded and securely stored in a sealed cupboard at CNRFP.

The full list of all the clinics (64) and villages (463) in the study area together with geographical coordinates was obtained with permission from the Burkina Faso National Institute of Statistics and Demography (*National Institute of statistics and demographic, unpublished data*). To calculate the pairwise distances between each patients’ village of origin and all the clinics in the study area, we used the package *igraph* [56] within the statistical software R [57]. We considered distances based on the road network, obtained from Open Street Map (www.openstreetmap.org) in a *shapefile* format. However, only major roads were available for our study area, so we digitized minor roads using QGIS [58] and digital images from Google maps (www.google.com/maps) (Fig. 1).

### Data analysis

We fitted the model (1) to the malaria case data using the four different approaches given by Eqs. (4–7), using Bayesian methods [59, 60] with the program JAGS [61], interfaced with R via the package *rjags* [62]. We used Markov Chain Monte Carlo (MCMC) algorithms (code provided in Appendix S1) to fit each of the three models to the distribution of road distance data (and age, sex and season, in case of Eqs. (5–7). We chose relatively non-informative priors for all parameters. For the coefficients $$a_{0}$$, $$a_{1} ,$$$$a_{2}$$, $$a_{3} ,$$$$a_{4}$$ and $$a_{5}$$ we chose diffuse normal priors centred at zero, corresponding to a null hypothesis of no-effect for each covariate. For the distance decay parameter $$c$$, we adopted a uniform prior with limits 0–1000 [63].

To achieve convergence, models were run for 10^5^ iterations. Means of posterior distributions with corresponding 95% credible intervals were obtained for all the parameters. We compared the four models using the deviance information criterion (DIC) [64]. Each model was also evaluated using a confusion matrix to compare the classification results (each case being assigned to one of the clinic, as predicted by the model) with the reference values (the true classification of each reported case, as observed from the data), calculating in particular the overall accuracy and the Kappa statistic of each model [65].

### Spatial mapping of reporting probability

Our best model was used to create a map of overall reporting probability as follows. First we created a square grid with 1 × 1 km resolution, then calculated the road-based distance between the centroid of each cell and all $N$ health centres in the study area (64). If the centroid was not on the road network, we created a further segment connecting it to the nearest stretch of road. Here, we are also taking into account the probability $P_{i,q}$ that the case goes completely unreported. For each cell of the grid we calculated the vector of probabilities $\varvec{P}_{i} = \left\{ {P_{i,1} , \ldots , P_{i,N} , P_{i,q} } \right\}$ of reporting at each of the $N$ clinics, according to: Eqs. (2) and (4). For such prediction, the formula used in Eq. (2) mirrors the one used for the model, however here we included the probability $P_{i,q}$ that the case goes l unreported, made in Eq. (2) assuming the form:

$$P_{i,1} = \frac{{g_{i,1} }}{{1 + \mathop \sum \nolimits_{n = 1}^{N} g_{i,n} }}$$

$$P_{i,2} = \frac{{g_{i,2} }}{{1 + \mathop \sum \nolimits_{n = 1}^{N} g_{i,n} }}...$$

$$P_{i,N} = \frac{{g_{i,N} }}{{1 + \mathop \sum \nolimits_{n = 1}^{N} g_{i,n} }}$$

8 $$P_{i,q} = 1 - \mathop \sum \limits_{n = 1}^{N} P_{i,n}$$

Conversely, we calculated the overall reporting probability in each cell $RP_{i}$ as.

9 $$RP_{i} = \mathop \sum \limits_{n = 1}^{N} P_{i,n}$$

and used this to produce a continuous surface of reporting probability (see Results and Fig. 4a). This map can be interpreted as a proxy for health care accessibility, as defined by product of different underlying processes (human behaviour, road network, and physical location of clinics). From this continuous surface we defined the catchment area of each clinic by assigning each cell to the clinic with the maximum probability of reporting. This included the category “un-reported”, if the probability of not-reporting $P_{i,q}$ was the relatively highest.

### Interactive mapping

To illustrate how our model can be used to explore its output under different scenarios of health centre distribution, we created a mapping tool that provides users with an interactive graphical user interface (GUI) using the packages *leaflet* [66] and *shiny* [67] in R. The GUI allows users to interrogate any point in space in terms of overall reporting probability, according to Eq. (9) and the catchment area to which it belongs. Moreover it allows the user to visualize the reporting probability of any clinic individually. Finally, we allow the user the option of selecting a subset of clinics to include or exclude from the model (i.e. can select from the $N$ clinics in Eq. (8)). In this way, the user can evaluate the contribution of each clinic in the public health network to case reporting and overall health centre accessibility, and highlight hotspots of un-covered areas under different and custom scenarios (for example in the case of one or more clinics being closed).

## Results

A total 59,822 individual cases of malaria reported at the 8 focal clinics were collated. Within this unprocessed dataset, the village of residence reported by patients couldn’t be linked to available data on community names (*National Institute of statistics and demographic, unpublished data*) in ~ 12% of occasions. As it was not possible to assign village-of-residence to these data, they were excluded from analysis. The final dataset thus consisted of 52,291 malaria cases, reported from 124 villages between January and December 2017.

**Fig. 2:**
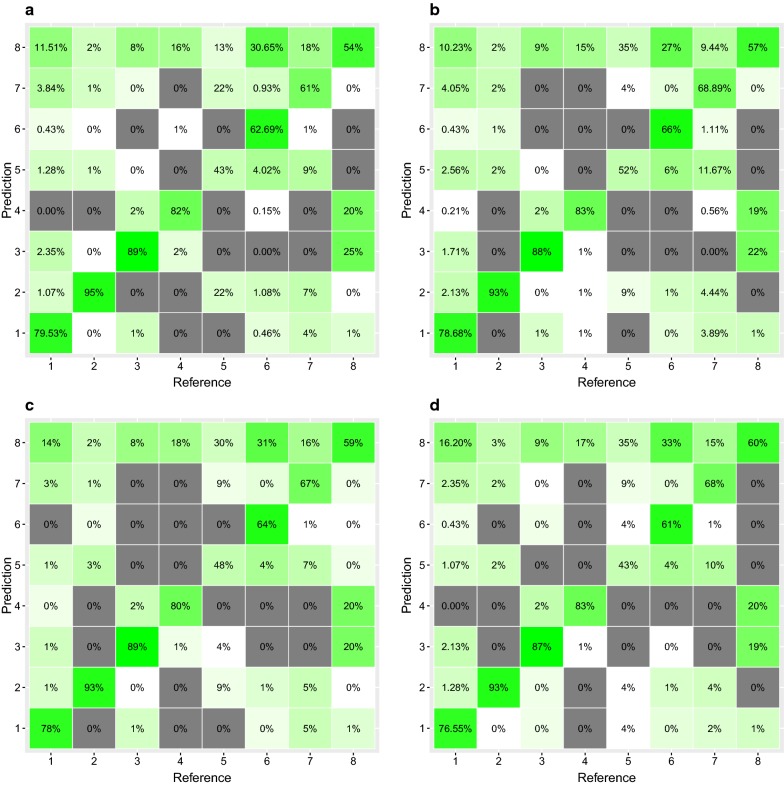
Confusion matrices for classification obtained with the multinomial models of reported malaria cases at 8 health centres (on x and y axes), as a function of distance from village of origin, in Komoé district, Burkina Faso. The diagonal elements represent the percentage of cases for which the predicted classification (case being assigned to a given clinic) is equal to the true classification, while off-diagonal elements are those that are mislabelled by the classifier. The predicted cases are obtained using **a** a model considering only the effect of distance, **b** a model considering the effect of distance and age, **c** a model considering the effect of distance and sex, **d** a model considering the effect of distance and season

MCMC for all four models reached convergence. The posteriors indicated that the probability of reporting a malaria case to a given health centre decreases with distance (Table 1). All of the models achieved a high overall accuracy, with percentages of correct classifications ranging between 73.7% and 75.0%, and high values of Kappa statistics (Table 1, Fig. 2). A random classification would correctly assign 12.5% of cases (1/8 clinics), this indicates that our model correctly classified 6 times more cases than a random classification. Among the four models, the best one, as shown by the lowest DIC value, considered only distance with no additional value from the covariates of patient sex or age or from the season. Furthermore, the posterior distribution of coefficients for age, sex and season, in models (5), (6) and (7) had credible intervals overlapping the 0 values, indicating lack of effect.

**Table 1:** Modified distance sampling models fitted to data of reported malaria cases at health centres in Komoé district, Burkina Faso

Model	$\varvec{a}_{0}$m (95% CI)	$\varvec{a}_{1}$ (95% CI)	$\varvec{a}_{2}$ (95% CI)	$\varvec{a}_{3}$ (95% CI)	$\varvec{c}$ (95% CI)	DIC	ΔDIC	Accuracy	Kappa
$g\left( d \right) = { \exp }\left( {a_{0} + a_{1} d^{c} } \right)$	14.77 (12.24/20.43)	9.17 (7.04/14.25)	–	–	0.18 (0.14/0.24)	3047.361	0.00	75.0%	0.70
$g\left( {d,A} \right) = { \exp }\left( {a_{0} + a_{1} d^{c} + a_{2} A + a_{3} Ad} \right)$	14.02 (11.86/19.09)	8.36 (6.64/12.90)	-8.02 × 10^−3^ (−1.95 × 10^−2^/3.48 × 10^−3^)	2.12 × 10^−4^ (−2.82 × 10^−4^/6.39 × 10^−4^)	0.20 (0.15/0.26)	3050.229	2.87	73.7%	0.69
$g\left( {d,S} \right) = { \exp }\left( {a_{0} + a_{1} d^{c} + a_{2} S + a_{3} Sd} \right)$	15.47 (12.39/25.68)	9.81 (7.19/17.53)	−0.08 (−0.42/0.26)	−6.44 × 10^−4^ (−0.01/0.01)	0.19 (0.11/0.24)	3051.809	4.45	74.7%	0.69
$g\left( {d,S} \right) = { \exp }\left( {a_{0} + a_{1} d^{c} + a_{2} R + a_{3} Rd} \right)$	18.12 (15.80/23.59)	12.35 (8.37/22.54)	−0.10 (−0.51/0.29)	−8.71 × 10^−3^ (−0.02/0.01)	0.16 (0.10/0.23)	3048.16	0.80	74.0%	0.69

The table shows the mean of posterior distribution with 95% credible intervals of the parameters for the multinomial process, deviance information criterion (DIC), overall accuracy (percentages of correct classifications) and Kappa statistics of each model

*d* distance from the health centre, *A* age of the patient, *S* sex of the patient (reference value: male), *R* season (reference value: dry season)

**Fig. 3:**
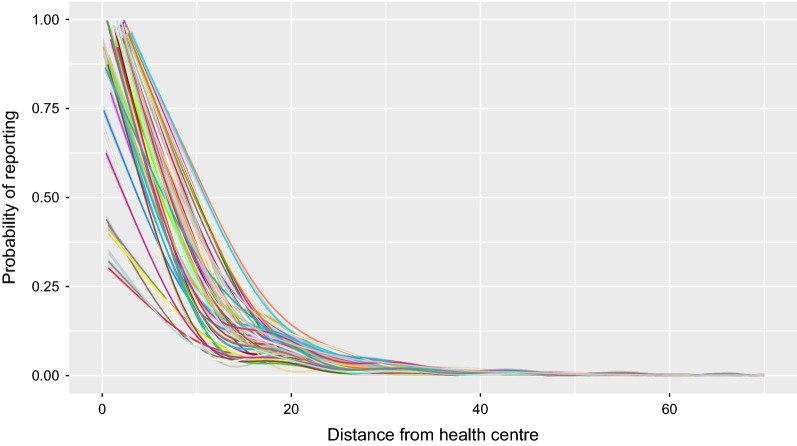
Predicted probability of reporting a malaria case to one of 64 clinics in Komoé district, Burkina Faso, as function of distance from village, based on actual reported cases. Each colour represents a health centre. Lines represent a smoothed conditional mean for each health centre

Once we applied Eqs. (4), (8) and (9) to each cell of the grid, we obtained the vector of probabilities of reporting to each of the 64 clinics in the study area. By plotting these probabilities against the distance from the clinic (Fig. 3), the probability of reporting a malaria case at a given health centre was estimated to be 1.0 for most of the cases when the patient lives at 0 km from a health centre. Nevertheless, for some clinics the probability was < 1.0 at zero distance, meaning that people living nearby a clinic may nevertheless report to another clinic that is sufficiently close. The rate at which the reporting probability drops with distance varies between clinics, taking values between 0.10 and 0.60 at 10 km, and 0.0 at distances higher than 30-40 km (Fig. 3).

**Fig. 4:**
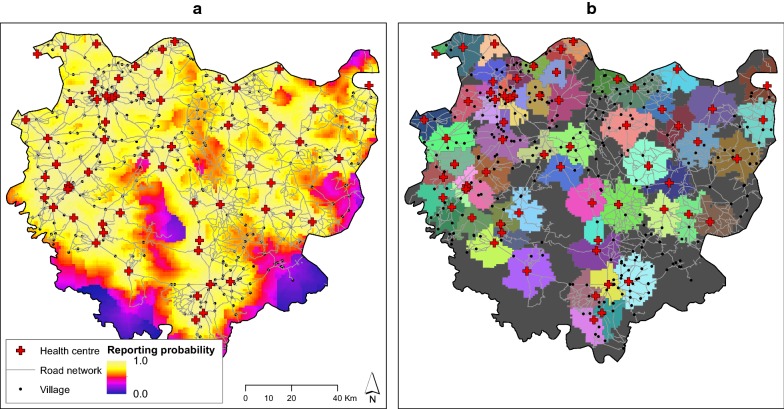
**a** Overall map of probability of reporting individual malaria cases and at health centres in Komoé district in Burkina Faso as predicted by the best distance sampling model and distribution of health centres (red crosses), villages (black dots) and road network (grey lines). **b** Catchment area of each health centre, defined by the model. Dark grey areas are cells in which the overall probability of not reporting is the relatively highest

Such values represent the probability that an individual malaria case would be reported at a single clinic, however, by combining the probabilities of reporting to all the clinics, and accounting for the probability of not reporting at all, we obtained the map of overall reporting probability and the map of catchment areas (Fig. 4).

**Fig. 5:**
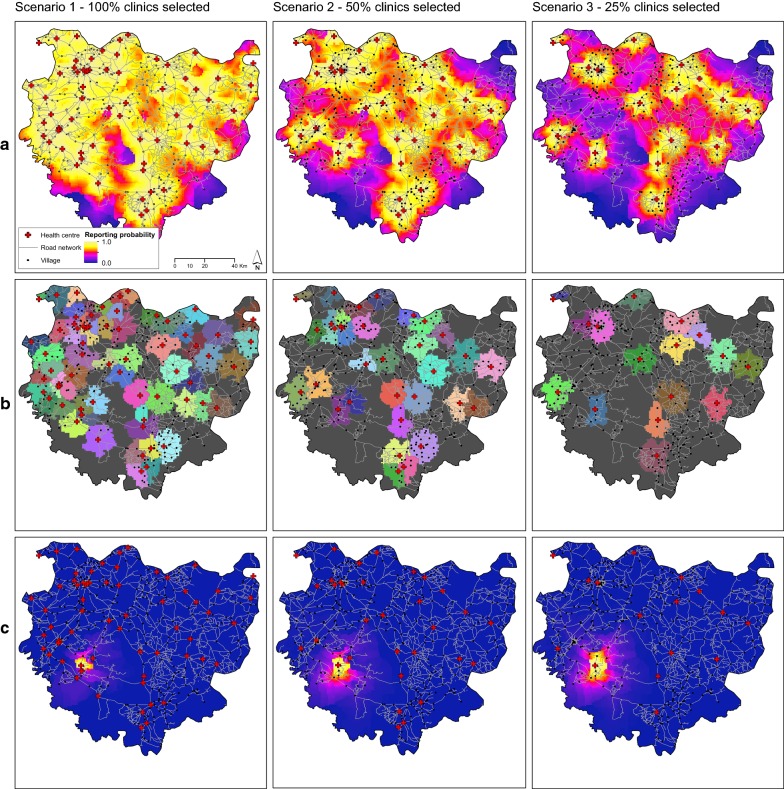
Examples overall reporting probability, catchment areas and reporting probability at a single clinic, according to 3 different scenarios of number and positions of health centres in Komoé district in Burkina Faso, as predicted by the best distance sampling model. **a** Overall reporting probability. **b** Catchment area of each health centre. **c** Reporting probability to an individual selected clinic

The interactive tool of predictive mapping can be found at http://boydorr.gla.ac.uk/lucanelli/distance_clinics_lite/. Although the inclusion of age, sex and season did not seem to improve the model, in the online supplements we present the results of the model including the season covariate, to provide an example on how this (or other covariates, depending on the specific study system) can be readily implemented in the predictive tool. Here, we provide three examples that can be obtained from this interactive tool (Fig. 5), showing the overall reporting probability (Fig. 5a), the catchment areas (Fig. 5b) and the contribution of a single given clinic in overall health network to case reporting (Fig. 5c). In particular, we show how these might vary according to different scenarios of subsets of clinics. In scenario 1, we show the maps when all the clinics are considered, in scenario 2 we considered a 50% random subset of clinics and in scenario 3 we selected a random 25% of the entire set of clinics. These examples illustrate how the overall probability of reporting decreases as clinics decrease and the catchment area and the relative probability of reporting to an individual clinic will increase.

## Discussion

Here, we have adapted a cornerstone analysis method from ecology, the point-transect distance sampling, to develop an innovative modelling framework to account for under-reporting bias in passive disease case detection. This quantitative tool uniquely accounts for the role of the observation process when predicting the spatial distribution of infection. Finally, we created a user-friendly predictive tool to explore how different scenarios of spatial allocation of health centres affect the probability of disease reporting.

Extending the point-transect distance sampling method from wildlife observation to disease reporting at clinics required some fundamental assumptions to be modified. Specifically, traditional distance sampling assumes perfect detection at zero distance and independence between observers. With the simplest form of point-transect distance sampling, double counts are allowed, because the probability of an object being recorded by one observer doesn’t affect the probability of the same object being recorded again by another. In our epidemiological system the events of interest are uniquely detected, meaning that the reporting clinic effectively “absorbs” the occurrence of the event, so that it is not reported elsewhere. By using the generalised formulation of the detection function presented in Eq. (4), and by formulating a multinomial process under the normalisation we are proposing, we could relax the first two assumptions, and allow clinics to have a detection probability lower than 1 even at zero distance, because it is possible that any given patient living in proximity of a clinic (d ≈ 0) might report an infection case to a different clinic, so that P(0) < 1.

Here, another difference from traditional point transect distance sampling methods is that the distances that drive the detection probability may not be Euclidean. In epidemiological applications, it is unlikely that human mobility would follow straight-line distances, so distances were calculated from the road network. Additionally, other travel covariates that may affect the probability of reporting would include road conditions or time of the year to allow for adverse road conditions during rainy seasons. Such information could improve the estimate of the rate at which reporting decreases.

Traditional distance sampling methods also assume that data are uniformly distributed around observation points. In wildlife surveys, if the animal population is not uniformly distributed (or if its distribution is unknown) this assumption can be met by ensuring that the observation points are positioned independently of the distribution of the study species. In the case of epidemiological data arising from passive surveillance, we violated this assumption because the observation points (health centres) are necessarily attached to the road network (i.e. villages and human settlements).

In traditional distance sampling, a monotonically decreasing shape of the detection function is generally assumed. In epidemiological applications, such monotonic behaviour is not certain because it could be confounded by topography (road network) and the relative location of other clinics that could give rise to multiple peaks the detection curves.

In our study, we modelled an infection reporting process as a function of distance from health centres [33, 36, 68–71], and showed how additional non-spatial covariates (e.g. age and sex of patient can be included) in the model. We did not find any clear effect of these demographic variables. However, as in traditional distance sampling, a better fit to the observed data might potentially be achieved if additional covariates are recorded and their effects on the detection function are modelled. Other non-spatial covariates known to influence malaria reporting include socioeconomic status [8, 10, 27, 28], ethnicity [29, 30], linguistic barriers [31], education [16, 17, 53, 54] and the severity of symptoms (e.g. asymptomatic cases [22–26]). All of these covariates, can be easily added to our basic model, as we demonstrated with age and sex.

Estimates of under-reporting generated here can be interpreted as the proportion of under-reporting due to distance to clinics (which sums to the proportion of under-reporting due to other non-spatial factors). Our framework however could be extended to account for asymptomatic cases by simultaneously using active surveys and passive case detection in a joint inferential framework [42].

Here, we assumed that the shape parameters of the detection functions were the same for each clinic. However, the shape of the detection function could be clinic-specific if, for example, people had a preference for some clinics over others that was unrelated to distance. Studies of malaria in Africa indicate that a range of variables influence a patient’s choice of clinic, including cost of services, opening hours, quality of infrastructure and equipment, attitude of staff and availability of drugs [17]. In other words, there could be a “clinic effect” on the detection function that could be modelled by including further covariates at the clinic level. Hospital type and diagnostic capabilities were homogeneous in our study system, but if any quantitative measure to distinguish one clinic from another are available, these can readily be included in the model.

In this study we did not explicitly consider the role of population size on malaria incidence quantification, but instead focussed only on modelling the probability of reporting. Therefore our study quantifies the probability that a case is reported at a given clinic, given that it arises at a particular point in space. This conditional model can be considered as the observation component in latent process model whose complementary part captures the epidemiological process generating disease cases in space. This could be modelled using a N-mixture model [63, 72]. In a previous paper [42], we proposed a framework for taking these two processes into account in an integrated model, simultaneously analysed data from active surveys and passive case detection, and validated it using a wide range of simulated scenarios. The results that we obtain on a set of real data here confirm that such methods provided powerful analysis tools for complex spatial epidemiology studies, and show promise for combining a spatially heterogeneous observation model with an epidemiological process in a novel inferential framework.

Although not sufficient on its own, the spatial access to health care is a necessary condition in the realization of actual access to health care [73]. Particularly in rural contexts of low-middle income countries, where health care planners must manage limited resources, there may be benefit from using strategic mapping tools to identify optimal location and distribution of clinics to maximize community access and uptake [38].

## Conclusions

Our proposed analytical framework and interactive tool allows to model different scenarios, in which the overall number and spatial distribution of health service provision can be varied, to assess the impact on people’s probability of reporting. Such information can be used to highlight therefore the hot-spots and cold-spots of health care coverage in the area, and it could enhance the decision-making processes with respect to planning new facilities. Furthermore, the possibility of mapping the catchment areas of each health centre provides a useful tool for evaluating the effectiveness of the current network of clinics and identifying which clinics are relatively under- or overexploited.

In its current form, our tool has mostly an illustrative purpose. However, if the shape of detection functions estimated here is transferable to other areas where no data are available, the same interactive tools can be applied just through provision of the GIS files (e.g. in a shapefile format) of health centres and the road network.

Several methods have been used to define the catchment areas of health centres [74]. Here we offer an empirical method, in the form of the interactive *Shiny* App, that can provide healthcare planners with a user-friendly tool to investigate the probability of utilization of a given health facility over a spatial gradient. This approach, by enabling visualization of how the health monitoring and treatment coverage are influenced by changing the number and position of health centres, will benefit infrastructure planning with respect to the positioning of new clinics, and new roads. Our framework can incorporate covariates at both the patient and clinic levels and has wider applicability beyond the specific disease, scale and covariate data available for our case study.

## Data Availability

The data that support the findings of this study are available from the first author Luca Nelli but restrictions apply to the availability of these data, which were used under license for the current study, and so are not publicly available. Data are however available from the authors upon reasonable request and with permission of the Burkina Faso Ministry of Health - National Centre of Research and Training on Malaria (*Centre National De Recherche et Formation sur le Paludisme*, CNRFP).
